# On the nature of real and perceived bias in the mainstream media

**DOI:** 10.1371/journal.pone.0193765

**Published:** 2018-03-23

**Authors:** Erick Elejalde, Leo Ferres, Eelco Herder

**Affiliations:** 1 Department of Computer Science, Faculty of Engineering, Universidad de Concepción, Concepción, Chile; 2 Institute of Data Science, Faculty of Engineering, Universidad del Desarrollo, Santiago, Chile; 3 Telefónica R&D, Santiago, Chile; 4 Institute for Computing and Information Sciences, Radboud Universiteit, Nijmegen, Netherlands; Consejo Nacional de Investigaciones Cientificas y Tecnicas, ARGENTINA

## Abstract

News consumers expect news outlets to be objective and balanced in their reports of events and opinions. However, there is a growing body of evidence of bias in the media caused by underlying political and socio-economic viewpoints. Previous studies have tried to classify the partiality of the media, but there is little work on *quantifying* it, and less still on the *nature* of this partiality. The vast amount of content published in social media enables us to quantify the inclination of the press to pre-defined sides of the socio-political spectrum. To describe such tendencies, we use tweets to automatically compute a news outlet’s political and socio-economic orientation. Results show that the media have a measurable bias, and illustrate this by showing the favoritism of Chilean media for the ruling political parties in the country. This favoritism becomes clearer as we empirically observe a shift in the position of the mass media when there is a change in government. Even though relative differences in bias between news outlets can be observed, public awareness of the bias of the media landscape as a whole appears to be limited by the political space defined by the news that we receive as a population. We found that the nature of the bias is reflected in the vocabulary used and the entities mentioned by different news outlets. A survey conducted among news consumers confirms that media bias has an impact on the coverage of controversial topics and that this is perceivable by the general audience. Having a more accurate method to measure and characterize media bias will help readers position outlets in the socio-economic landscape, even when a (sometimes opposite) self-declared position is stated. This will empower readers to better reflect on the content provided by their news outlets of choice.

## Introduction

The media have a strong influence on how people perceive the world that surrounds them. More and more power has been ascribed to the modern press since its inception, even calling it the “Fourth Estate” [1] emphasizing its independence and its ability to provide strict limits to what governments may or may not do. There are well known examples of the press even toppling governments: the Washington Post in the Watergate scandal is perhaps the most resounding example.

However, as the media grows in power, the political and economic interests of news outlets and the ones who control it have grown as well, which has its impact on the news that the population of a territory gets served. Among others Herman and Chomsky [2] argue that political and doctrinal interests have penetrated the press at different stages of the news generation process, deliberately or accidental—for example through homophily effects. In certain cases the resulting bias is explicitly stated, in other cases—like FOX News—the bias is known but not explicitly communicated. People usually have some intuition of media bias. For average readers, though, it is very difficult and time-consuming to be aware or even find the bias of all media outlets, let alone quantify these biases and give them a total order in terms of the magnitude of the leaning.

Bias in the media is a global phenomenon, not exclusive to one kind of economy or particular political system. As such, there is now a quickly growing body of empirical evidence on its existence [3–5]. In previous work [6], we showed several types of bias in media coverage of ongoing news stories on crises in the world. What has not been studied as deeply, however, at least not quantitatively, is *how* outlets could be positioned in a socio-economic space. Knowing *the nature* of media bias will help individuals and organizations take actions that counteract bias. If, for example, a newspaper claims to be objective, but is in fact “right-wing, conservative” (as is the case with El Mercurio in Chile [7]), people should be able to recognize this and take this bias into account when reading its content. The case of El Mercurio is quite clear, and being a very old, traditional newspaper, the bias is actually known and arguably accepted. It is important to emphasize here that “bias” is not categorical, but comes embedded in a geopolitical news context determined by other outlets in the region [8]. In other, bolder words, some bias is inherent to all media, but how biased they are, depends, to an extent, upon a comparison to other media.

In this work, we automatically identify the (largely implicit) socio-economic “relative bias” of news outlets in the context of Chilean media. The value of our methodology and study here is to position those media outlets that do **not** state their socio-economic bias, or are not even aware of their bias. Socio-economic studies at this scale may help uncover patterns of editorial policies that show a systematic bias that favors governments’ propaganda or private economic interests over social welfare. Operationalizing bias is a difficult task. It relies not only on linguistic information, but also on the actual geo-socio-economic, and even historical, context of the newspaper. We propose to automatically categorize news outlets by analyzing what they “think” about certain relevant, controversial topics using their tweet content and then map these worldviews onto a well-known political quiz: “The World’s Smallest Political Quiz” (henceforth *PolQuiz*) [9].

The *PolQuiz* has ten question, and it was originally intended for an American audience. Although we believe this does not imply a loss of generality wrt Latin American culture, at least in the topics chosen. It does, obviously, impact the polarity of attitudes towards those topics, but that is what we explore in these pages. It was designed by the Libertarian Advocates for Self Government [10], created by Marshall Fritz in 1985. The quiz is based on the one proposed by David Nolan Chart in 1971 [11], which in turn can be traced back to a 2D chart proposed in 1968 [12], representing variations in political and socio-economic orientation.

In short, we use what the media say on Twitter to position them in a Cartesian plane that tells us more about their orientation based on Fritz’ quiz. In turn, the *PolQuiz* results motivate a deeper investigation into the nature of the found bias, which we study through the vocabulary used and entities covered by the news outlets. Finally, we conducted a survey that confirm that media bias has a noticeable impact on how news related to controversial topics are presented.

## Related work

There are several works related to the topic of media bias [5, 13–16]. Some works do not try to identify bias directly, but instead try to identify and track events in order to present different points of view of the same affair to the readers in order to counteract these possible bias [13]. These are complemented by works like J. An *et.al*. [17], which create a so-called landscape of newspapers based on the similarity of their communities. They measure the exposure of Twitter users to politically diverse news. Other authors assume a certain leaning by contacts association [18]. In [4] the authors go deeper and try to identify different kinds of bias, what they term gatekeeping, coverage and statement bias, according to the stage at which the news acquire the alleged bias.

Most outlets identify themselves as unbiased free press, which makes the discussion on the direction and degree of media bias very controversial. To be fair, it is true that “bias” in journalism may arise naturally out of the interaction of reporters, rather than *a prior*, but this discussion is left for another paper. Media bias is usually found in the editorial policies that ultimately decide which stories are worth publishing and which amount and angle of coverage they get [4, 13, 19].

This bias reflects the political and socio-economic views of the institution, rather than the point of view of a particular reporter. For example, the authors in [2] use a few recent events to point out how the press applies the word “genocide” to cases of victimization in non-allied states, but almost never to similar or worse cases committed by the home state or allied regimes. In the latter case, they could use terms such as “repression of insurgency”.

In [20], the authors defined a model to predict political preference among Twitter users. Through this model they calculate, for each user, a ranking of the likelihood that they prefer a political party over another. This model is based on the usage of *weighted words*. The words and their weights are extracted from tweets of candidates of certain political parties. Using these weights, in combination with Twitter specific features (retweets, following, etc.) the authors train classifiers that achieve a performance similar to that of human annotators. Similarly, in [14], the authors estimate the bias in newspapers according to how similar the language is to that used by congressmen for which a right/left stand is known. One interesting result is that bias in the news is found to be correlated to political inclinations of readers, showing a tendency in these news outlets to maximize profit by “catering” to a certain audience.

The topology of the social network on its own has also been shown to give enough information to create classifiers concerning a user’s preference, even when the choices are very similar [21](e.g. Pepsi vs. Coca Cola, Hertz vs. Avis or McDonalds vs. BurgerKing). Although we carefully select the dataset to use in our experiments to achieve extensible results [22], we notice here that in our dataset, news outlets (which may be considered the participants of our studies), regularly talk about these controversial topics, and thus, it is possible to use traditional methods to find a political stand.

Combining topological characteristics of the social networks with language features has also been tested [18], showing that users tend to interact more frequently with like-minded people. This phenomenon is known as *homophily*. As we mentioned before, our dataset is derived from a special type of users (news outlets Twitter accounts), and this method may not apply directly.

As an alternative approach, in [15] the authors propose a semi-supervised classifier for detecting political preference. They design a propagation method that, starting with a few labeled items and users, creates a graph representing the connections between users and items or even users with other users. Based on the same phenomena of homophily, they assume that users interacting with the same item, or with each other, most likely have the same political leaning. This way, they can propagate the labels from tagged users and tagged items to the rest of the graph. They report that the system achieves over 95% precision in cross-validation. In [16, 23], the authors also follow a propagation strategy to compute the political preference of Twitter users, but using Congress members as the initially tagged users.

In [24], the authors describe a framework to discover and track controversial topics that involve opposing views. They first use tags that represent each side (e.g. “#prolife”—“#prochoice”) as seeds to find an expanded set of labels to represent each side. This may also help in cases where labels may change over time as the result of new arguments for either side. With these sets of labels they identify strong partisans (anchors) that have a clear lean to one side. Having these anchors and a graph representing relationships between users (based on similarity of tweet content or based on re-tweets), they propagate the classification through the graph inferring the opinion bias of “regular” users.

Yet another approach to quantifying political leaning is presented in [3]. They based their analysis on the number of tweets and re-tweets generated about different political events associated with some predefined topics. The authors developed a model that takes into account both the sentiment analysis of the tweets and the number of time they are re-tweeted to calculate the political leaning score of each outlet.

In [8], the authors propose an unsupervised model based on how news outlets quoted president Barack Obama’s speeches. The findings suggest that quotation patterns do reveal some underlying structure in the media, and that these may be evidence of bias. They found that one of the identified dimensions roughly aligns with the traditional left(liberal)-right(conservative) political classification and the other with a mainstream/independent one. This is a strong finding. Still, we believe this is to be somewhat expected, given the selected corpus; namely, presidential speeches in the strongly bipartisan system that dominates U.S. politics. Although this model helps classify and quantify bias in the media, it does not explain the causes and nature of this bias.

In this paper, we present a new methodology that quantifies the political leaning of news outlets based on the automation of a well known political quiz. The prediction of the answers for each question for each outlet is generated based on the polarity of their tweets on subjects related to the issues addressed in the quiz. The automation of a quiz has been used before to automatically classify mood [25] but, as far as we know, this is the first attempt to quantify media bias using this approach. We focus on Chile as a case study because most of the literature report only on Twitter content from English-speaking countries, which may bias the knowledge we posses in general about these issues.

## Methodology

In this section, we describe our dataset, followed by an overview of the *PolQuiz* and an explanation on how we applied this quiz to our data. In Section Rank difference, we introduce the Rank Difference method for investigating the nature of bias. We conclude with an overview of the survey that we carried out to measure perceived bias.

### Data

Every news outlet has some presence on the web, which opens the possibility for the automatic collection of the news stream they produce. Twitter, an online social network that enables users to send and read short messages called “tweets”, is a prime example of a web platform that allows this, and Chile ranks among the top-10 countries regarding the average number of Twitter users per 1000 individuals [26]. Twitter offers an open API to automatically access the flow of tweets and query the system for user profiles, followers and tweeting history. This makes it possible to explore the behavior and interactions of personal and institutional accounts, developing and testing social theories at a scale never seen before. This is the closest thing we have to a record of the every-day life of over 300 million people (Twitter reported 328 million monthly active users in the first quarter of 2017 [27]). We treat every tweet as an independent document from which we can extract a statement. We assume that these reflect the ideology of the news outlet as an entity. As many others, we use Twitter as our source documents to study news [3, 28, 29]. Twitter and other social media have become hubs of news for an increasing number of users [30]. A tweet from a media outlet is a man-made summary of the news, usually in the form of a *headline*. It conveys the main idea, and hence arguably the main editorial point of view. Headlines of online news articles have shown to be slightly more reliable than full text for adequately providing a high-level overview of the news events [31–33]. These summaries are expected to be representative of the newspaper’s bias [34, 35], but with the advantage that bias is easier to detect than in a full articles (shorter, to the point), given the heterogeneity of sites (not all provide RSSs and we would have to rely on scraping and more sophisticated natural language processing tools, to detect topics, sponsored news, etc.). Tweets also contain features/annotations (e.g. hashtags (#) and mentions (@)) that help to give semantic to the text. Twitter “texts” are concise, the interface much richer than scraping (we, for example, don’t “miss” tweets, and get much more metadata from Twitter (timestamps, number of retweets, likes, etc.) which must be found and “parsed” in free-text articles, the editorializing is arguably stronger, NLP techniques need not be as sophisticated and simple n-grams will do. Because of this, tweets, in all their simplicity, seem to be not only enough, but a better fit, for our profiling purposes.

To create our database of outlets, we used different sources, with Poderopedia’s “influence” database [36] as our baseline, manually adding other news outlets in Chile. Our database contains 399 *active* accounts. An account is considered *active* if it tweets at least once a month. The data set contains 1,916,709 tweets, spanning a period of 8 months—from October 6, 2015 to June 4, 2016. The accounts vary dramatically in tweet publication behavior, with some having published more than a hundred thousand tweets to others with less than a hundred in this timeframe. Out of the 399 active accounts, only 269 outlets published at least one document about the topics of interest.

### PolQuiz

The *PolQuiz* has ten questions, divided into two groups: economic and personal issues, of five questions each. The answers to the questions may be *Agree*, *Maybe (or Don’t Know)* or *Disagree*.

**Personal issue questions:**
1 Government should not censor speech, press, media or Internet.
2 Military service should be voluntary. There should be no draft.
3 There should be no laws regarding sex between consenting adults.
4 Repeal laws prohibiting adult possession and use of drugs.
5 There should be no National ID card.

**Economic issue questions:**
6 End corporate welfare. No government handouts to business.
7 End government barriers to international free trade.
8 Let people control their own retirement: privatize Social Security.
9 Replace government welfare with private charity.
10 Cut taxes and government spending by 50% or more.

Based on the answers to these questions, the quiz-taker is classified into one of the five categories: left-liberal, libertarian, centrist, right-conservative, or statist. *Left-liberalism* is a political ideology that supports governments that take care of the welfare of vulnerable people and keeps a centralized economy, but at the same time, allows a great deal of liberties in personal matters. *Libertarians* seek freedom in both economic and personal issues, minimizing the role of the state in all matters. An extreme position in this direction would be anarchism. On the other side, *statists*—or supporters of a big government—want the state to regulate both personal and economic issues. Examples of this position would be totalitarian regimes, such as Kim Jong-Un in North Korea. *Right-wing conservatives* are more reluctant to accept changes in personal issues and want official standards on these matters (i.e. morality and family traditions), but demand economic freedom and a free market. Finally, *centrists* accept or even support a balance between the government reach and personal/economic freedom. They favor selective government interventions to current problems while avoiding drastic measures that may shift society to either side of the spectrum.

For each *Agree* answer, we increase the score of the quiz-taker in the corresponding dimension by 20 points. If the answer is *Maybe (or don’t know)*, we only add 10 points. Finally, if the answer is *Disagree*, no points are added. This way, if the quiz taker agrees with all the issues in one dimension, it will be in one end of that axis. In the other extreme of the axis, we will have a quiz-taker who disagrees with all issues in that dimension. In our study, we assume that news outlets are (or strive to be) unbiased, so in an ideal world, most of their comments should have no polarity toward any side of the issue and, as such, they should score as a *Maybe*. Another expected behavior would be that news outlets report on both sides of the issue to cover different points of view. Both approaches would result in the news outlet being in the center of the graph.

There is a long tradition of surveys to profile individuals and position them on a socio-economic landscape (see, for example, [37]). The instrument we use here, “The World’s Smallest Political Quiz” (PolQuiz), follows this tradition. The theoretical foundations of the *PolQuiz* can be traced back to the works of David Nolan, Maurice Bryson and other political scientists in the late 60s and early 70s [11, 12]. Although there are other more “complete” quizzes online (see, for example, *The Political Compass* [38]), an advantage of the *PolQuiz* is that it is “open source”, in the sense that the scoring system is known, unlike for example the Political Compass one mentioned above. It is also short (only five questions per dimension), very popular (the Advocates for Self-Government (founded by the creator of the quiz: Marshall Fritz) claims that the quiz has been taken online more than 23 million times [39], and it has been used, evaluated and cited scientifically [40, 41].

### Operationalizing the Quiz

We filtered the collected tweets to get only those with information regarding the issues referred to in the *PolQuiz*. For this, we created a seed query for each question, containing a set of preselected keywords (see Table 1).

**Table 1:** Initial set of keywords for each query.

Question	Keywords
q1	(censura—libertad) & (prensa—discurso—expresion)
q2	(servicio—reclutamiento—entrenamiento—reserva) & (militar—ejercito—armada)
q3	(ley—legal—legislacion—regulacion—penalizacion) & (sexual—prostitucion—sexo—sodomia—gay) & ¬(infantil—menor—niño—acoso—abuso—agresion)
q4	(ley—legal—legislacion—regulacion—penalizacion) & (droga—marihuana—cannabis—psicotropico—cocaina)
q5	inmigracion—inmigrante—refugiado—xenofobia
q6	(subsidio—bienestar—ayuda) & (corporativa—empresa)
q7	(trato—tratado—convenio—negociacion—relacion) & (comercial—economica) & (internacional—bilateral—gobierno—libre—liberal—barrera—proteccion—bloque)
q8	(“seguridad social”—afp—pension—jubilado—prevision) & (privada—gobierno—estatal)
q9	(“beneficio sociale”—bono—“ayuda sociale”—“programa social”) & (gobierno)
q10	(reducion—recorte—aumento—incremento) & (impuesto—gasto) & (gobierno—gubernamental)

Our actual queries are designed so they can also find variations of the keywords (such as variations in gender and number). AFP stands for Administradoras de Fondos de Pensiones (Chilean pension system)

With the subset of documents returned by the seed queries, we then analyzed the hash-tags to find an expanded set of labels that may represent related aspects of the same issues [24]. We removed hash-tags that contain the name of a news outlet, as it is common practice in newspapers accounts to use hash-tags to refer to themselves or the original source of the news (regardless of the subject). We also remove hash-tags with names of politicians: even when these politicians could potentially provide some relevant documents, they also introduce a lot of noise, mostly due to the salience of politicians who appear regularly in the news for a wide variety of issues not necessarily related to the query in question. The new labels are added as keywords to the original query. Our enriched queries give us the final set of tweets used to evaluate any possible bias of each news outlet, see Table 2.

**Table 2:** Tweets extracted from our corpus after applying the enriched queries.

Qs	tweets	Training set (TS)	% Agr (TS)	% Mb (TS)	% Dis (TS)	% Not rel (TS)	Prc (±2 * *stdev*)
q1	374	179	48.6	16.7	17.8	16.7	0.76 (± 0.14)
q2	194	132	18.1	29.5	20.4	31.8	0.87 (± 0.11)
q3	144	78	57.6	05.1	24.3	12.8	0.83 (± 0.17)
q4	597	203	61.0	08.3	14.2	16.2	0.80 (± 0.10)
q5	746	219	35.1	12.7	15.9	36.0	0.73 (± 0.16)
q6	636	117	26.4	25.6	23.9	23.9	0.53 (± 0.20)
q7	1162	238	29.8	24.7	39.9	05.4	0.76 (± 0.09)
q8	251	117	21.3	09.4	41.8	27.3	0.76 (± 0.13)
q9	298	167	05.9	13.1	69.4	11.3	0.87 (± 0.09)
q10	8573	466	16.7	13.3	66.0	03.8	0.71 (± 0.06)

The last column indicates the average precision obtained by the model in cross-validation (See Section Operationalizing the Quiz)

Having the set of tweets for each question, we classified their polarity *with respect to the corresponding question*. For example, for *question 7* (**q7**), a tweet classified as *Agree* is *“TPP abrira puertas a más de 1.600 productos chilenos no incluidos en acuerdos vigentes.” (tr. TPP will open doors for more than 1.600 Chilean products not included in existing agreements)*. For that same question, the following tweet disagrees with it: *“El TPP: un misil contra la soberanía” (tr. TPP: a missile against our sovereignty)*. In other words, we classify the polarity of the tweet with respect to the corresponding issue. As the number of tweets is too large to label manually, we created and trained a supervised model for each question. This approach also allows us to scale in the presence of an even larger number of resulting documents.

To create a representative sample for the training set, we randomly select, where possible, two tweets from each question from each news outlet. We took care to not include duplicate tweets (tweets with the exact same text) published by the same outlet. The training set consisted of 1916 documents (an average of about 190 documents per question). We manually classified this training set in four groups: *Agree*, *Maybe*, *Disagree* and *Out of topic (Not relevant)*. The distribution of each training set is shown in Table 2.

For the automatic classification task, we used a “Randomized Trees” model [42] (Implemented in the python library scikit-learn in the module sklearn.tree.ExtraTreeClassifier). Decision trees are less susceptible to overfitting, considering that we have relatively small training sets. Given that the classes in our training set are not evenly populated, we decided to evaluate the model using a 10 iterations stratified shuffle-split cross validation. Each fold leaves out 20% for validation. The other 80% is selected while preserving the percentage of samples for each class. The accuracy values for each model is presented in Table 2.

After the classification stage, we scored each news outlet on each question. We removed those documents classified as *off-topic (Not relevant)*. We scored the remaining documents’ polarity according to the *PolQuiz* scoring system and we found the average for each question/news-outlet pair. For simplicity, in the question/news-outlet pairs for which we have no associated documents, we assume a *Maybe (or don’t know)* answer. This assumption is the least disruptive towards the default supposition of an unbiased media.

In order to find out how sensitive the observed bias is to noise, we repeated the scoring steps 20 times. Each time we leave out 5% of the tweets, selected at random while maintaining the original distribution of documents per question. Each time we measure the average score of the news outlets for which we were able to answer at least one question in the corresponding dimension. We did not go over 5%, because the smallest news outlets already have a small set of documents: removing too many entries would have resulted in the elimination of an entire outlet, affecting the results.

Finally, we tested how the entire system adapts to the local environment. For a proof of concept, we introduced the subject of abortion in the personal dimension. This topic appears among the personal issues in other political quizzes (e.g., *The Political Compass* [38]) In addition, abortion was a very relevant and controversial topic in the Chilean media during this period because of a new bill presented by the president and approved by the Chamber of Deputies to legalize the abortion on three grounds: pregnancy resulting from rape, lethal fetal infeasibility or danger to the life of the pregnant women.

We formulated this new question as follows:

0. All women should be free to choose whether she wants to terminate her pregnancy or not.

Notice that the question is formulated in the same “direction” as the rest of the questions. This is, agreeing with the statement will be an indicator of a more liberal tendency by the quiz taker.

We apply the same methodology described before to the original *PolQuiz*. We named this question **q0** and the query we applied (before injecting the hash-tags) is shown in Table 3. The enriched query returned 4891 documents from our corpus. We selected two random documents for each news outlets to create a training set containing 409 tweets. We had an average precision of 0.70 (±0.08) in the 10 iterations stratified shuffle-split cross validation.

**Table 3:** Initial set of keywords for q0 query.

Question	Keywords
q0	(ley—legal—legislacion—regulacion—penalizacion—despenalizacion) & (aborto—interrupcion—embarazo)

Our actual queries are designed so they can also find variation of the keywords (such as variations in gender and number)

### Rank difference

Using the *PolQuiz*, we aim to show empirically that the news media in Chile have some socio-economic leaning. This means that news outlets tend to have a stand in at least some of the controversial topics that dominate the political landscape of the country. However, we are also interested in the *nature* of the bias regarding such controversial topics.

To do this, we use the *rank difference* method proposed in [43]. Rank difference is used to identify terms that characterize a specific domain. For example, the word *court* will be probably identified as a term if we are analyzing a corpus of legal documents. The method creates a ranking of words based on their frequency in a domain and a generic corpus. By comparing their relative position in both corpora, the algorithm identifies words that are significantly more used in a given domain. These unusual word frequencies are used as an indication of the importance of these words in the given domain. The formula for calculating rank difference is shown in Eq 1,

$$\tau \left(w\right)=\frac{r_{D}\left(w\right)}{\sum_{w^{\prime }\in V_{D}}r_{D}\left(w^{\prime }\right)}-\frac{r_{B}\left(w\right)}{\sum_{w^{\prime }\in V_{B}}r_{B}\left(w^{\prime }\right)}$$ (1)

where *r*_*D*_(*w*) and *r*_*B*_(*w*) are the ranks of word *w* in the domain and background corpus respectively. Rank normalization is done against the summation of all word rankings in the corresponding vocabulary (*V*_*D*_ and *V*_*B*_).

### Survey

To investigate to what extent the bias—as measured with the *PolQuiz* and investigated using the rank difference method—is perceived by the general audience, we conducted an online survey. We chose abortion as the topic of this survey, as this is (as explained in Section Operationalizing the Quiz) a current and controversial item in Chile that has received an important amount of coverage in the local media. This means that most people in Chile are aware of the discussion and probably have their own opinions. We also restricted our survey to the subset of news outlets who had relevant tweets for at least four questions per dimension (see Section Results) since these are the ones that we were able to position in the chart with the highest confidence.

We calculated the bi-grams’ rank difference (see Section Rank difference) for each news outlet. We decided to present bi-grams to users in the survey instead of words, because bi-grams offer more context, so it was easier for people to assess the connotation of a word or set of words within the selected topic. We also decided to use bi-grams over named entities because people not always recognize all the names involved in the discussion, although they do have an intuition in the discourse and the arguments used on both sides.

For each survey we presented a randomly selected and anonymised list (each list represents a news outlet) with the top-20 ranked bi-grams in one column and the bottom-20 bi-grams in another column. The top-20 list was presented as the words used with a relatively high frequency by one outlet. The bottom-20 list was presented as words the outlet tried to avoid or used with a relatively low frequency. The user had to answer if, based on these lists, he or she considered the outlet to be “in favor” or “against” abortion. The user could also respond with an “I can’t tell” option. A user could answer the survey more than once, but the random selection was always made from the remaining lists.

We scored the “perceived bias” for each news outlet based on the answers we received in the survey. For each outlet, we calculated the percentage of users that answered “in favor” and subtracted the percentage of “against” answers. These percentage include the users that answered “I can’t tell”. So, we consider outlets with a negative score to have a conservative “perceived bias”. Equivalently, outlets with a positive score are considered as liberals in our “perceived bias”. It is worth noticing that an unbiased news outlet should be expected to score close to zero (because it should have mixed signals and, either a proportional number of user labeled in each direction or most users were unable to classify it).

## Results

In this section, we first show how the *PolQuiz* helps to measure the bias in the media. We investigated the benefit of contextualizing the quiz by transforming the political space and by including new questions that fit the current political landscape in Chile. We verified that our results are stable to small changes in the dataset and we explore the nature of the bias showed by the media by using the rank difference method. We show the differences in the type of coverage between news outlets of various leanings when we deep in the analysis of one particular topic. We also investigate, using a survey, to what extent this bias is perceived by the general audience. Finally, we show empirical evidence of the existence of media capture.

To summarize the results, we show that the media landscape in Chile in terms of absolute positions is highly in line with the political orientation of the government. Within this landscape, relative differences in biases can be observed, which are in line with public perception—as captured by tendencies reported in Wikipedia. Further, we show that the nature of the bias can be explained and shown by the entities and sentiment related to the news outlet. Finally, we discuss how the media landscape, in terms of absolute positions, shifted along with a shift of the government.

### Measuring bias using the PolQuiz

Our list of news media covers outlets of very different sizes (as measured by number of followers of that outlets’s Twitter account). This difference in size is also reflected in the number of documents related to the issues in the *PolQuiz* that we are able to retrieve for each news entity. We found that, in general terms, the larger the outlet (measured in number of followers), the more they talk about (at least) the socioeconomic and political issues related to the *PolQuiz*. Likewise, mid-size outlets are well represented and show an active participation in the selected topics. In this work we are interested in the behaviour and bias displayed by each different outlet in the media landscape, regardless of the size. This notwithstanding, we wanted to make sure we were covering the entire spectrum of the Chilean media. Our methodology is able to find the outlet’s position even with just a few tweets. The evident extension of a more general analysis that takes into account the weight that each news outlet contributes to the global media bias is left for future work.

#### Absolute bias: The media landscape

**Fig 1:**
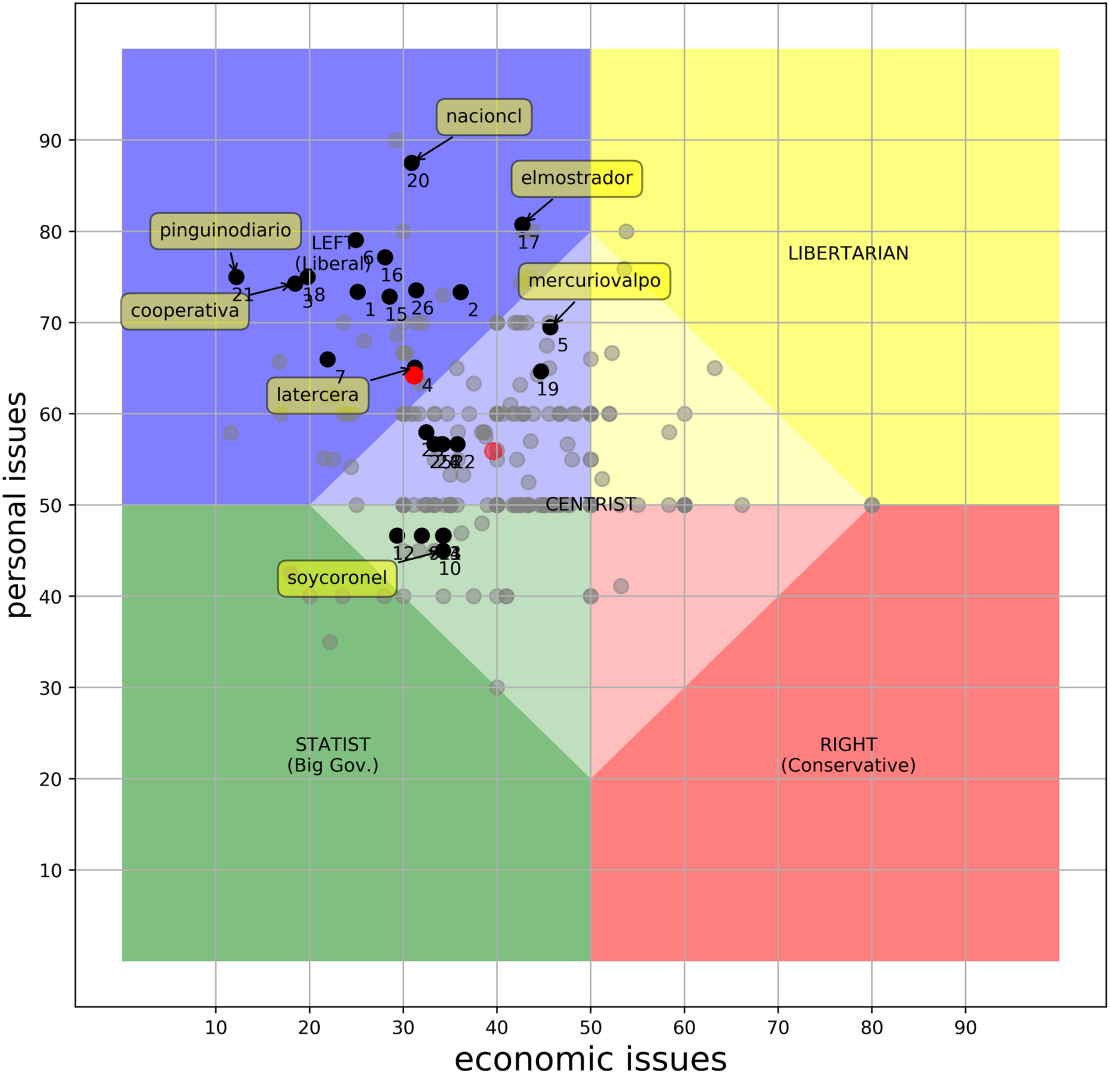
Absolute positions of the Chilean media. The chart shows the position of the outlets for which we were able to answer at least one question. Dark dots represent 26 news outlets who had relevant tweets for at least four questions per dimension (the *26ers*). The solid red dot shows the average position of the *26ers*. 1. adnradiochile, 2. biobio, 3. cooperativa, 4. latercera, 5. mercuriovalpo, 6. publimetrochile, 7. emol, 8. soyarauco, 9 soyconcepcion, 10. soycoronel, 11. soyquillota, 12. soysanantonio, 13. soytalcahuano, 14 soytome, 15. dfinanciero, 16. el_ciudadano, 17. elmostrador, 18. tele13_radio, 19. el_dinamo, 20. nacioncl, 21. pinguinodiario, 22. soychillan, 23. soycopiapo, 24. soyvaldiviacl, 25. soyvalparaiso, 26. t13.

To understand the results, a few preliminaries about the Chilean context is in order. First, the current president (as of June 2017), Michelle Bachelet, is affiliated with the socialist party. In general, the ruling coalition is “Nueva Mayoría”, which mainly consists of center-left to left-wing parties. Second, one of the strongest component in this coalition is the Christian Democratic Party. Christian democracy is still a center-left political ideology, but probably the most conservative within the government, especially in personal issues. With this in mind, Fig 1 shows the absolute positions of 269 news outlets that published at least one tweet related to the issues of at least one of the questions of the *PolQuiz*. Black dots identify those outlets for which there are answers for at least four questions on each dimension. Those which do not fulfill this criterion, but for which there is still some information, are identified as gray dots. Notice that for outlets with no information on a given question we assumed, conservatively, that they were “unbiased”, in the sense that they did not explicitly pronounce their stance one way or the other. We can see that outlets tend to tweet more content pertaining to the economic axis than to the personal axis. This may suggest that communicating economic issues is more important to the news system in terms of reaching or influencing their audience. A drastic shift in economic issues may invoke fear of losing your job or livelihood. Meanwhile, personal issues like freedom of speech are more ideological but of less immediate effect. The figure also shows that there is a clear preference in the Chilean media for the *left-liberal* end of the spectrum. This is explained, at least in part, by the political context of Chile during the observed period, discussed above. So, in this case, the observed leaning of the media has a similar tendency to the political alignment of the ruling coalition. This result also lends some evidence to the *Propaganda Model* [2]. This model proposes the existence of implicit filters that allow the political and economic elites to mold the content of the media to benefit their private interests. We explore more in how a change in government may influence the media position in Section *The influence of government orientation on the media landscape*. Notice that this tendency also coincides with the “liberal media” label that is frequently used, implying a popularly perceived Left-liberal leaning in the majority of the outlets [44].

Of the 269 news outlets, our method yielded 26 that answered at least four questions on each dimension (we will call this subset of news outlets the ***26ers***). This represents 10% of our database and 13% of those that regularly report on economics and politics. The ***26ers*** account for the 45% of the tweets relevant to the subjects in the *PolQuiz*. In Fig 1 we explicitly labeled some of the most prominent ones to help understand the general landscape.

#### Measuring perceived bias

We wanted to investigate if the outlets’ bias obtained using the *PolQuiz* corresponded with the popular perception of their political orientation. We annotated the ***26ers*** political alignment using information extracted from Wikipedia, the official web site of the news outlet or the political alignment known for their owners. Since Wikipedia pages are crowdsourced content, we consider the political alignment extracted from there as either self-declared or a popular perception. Remember that the Christian Democratic Party is part of the center-left coalition that was ruling in Chile during the observed period, and was generally in favor of the social changes promoted by the government. We used the label “Christian democracy” to group the outlets associated to this party, and assigned them a left-leaning position in our analysis. This classification is taken as ground-truth to evaluate our model.

There are three of the ***26ers*** for which we could not find reliable information on their political leaning. Two of them (tele13_radio and t13) are owned by Chile’s Grupo Luksic (one of the richest families in Chile and Latin America). The third one (pinguinodiario) is a mid-size daily local newspaper headquartered in Punta Arenas, in the south of Chile. In the ***26ers*** there are also two outlets that belong to international groups originating outside of Chile: publimetrochile and adnradiochile. The first one is owned by Metro International, a Swedish global media company based in Luxembourg that publishes the Metro newspapers in many big cities around the world. The other one is controlled by a subsidiary of the Spanish group PRISA. Although these international companies may also have economic interest in the country that motivates a political leaning, it is probably less influential in theirs outlets editorial policy. We did not make a bias inference either for these cases. Nevertheless, very much as for any other outlets outside the ***26ers***, some valuable information can be learn from their automatic classification.

In the rightmost of the group, we have mercuriovalpo (Tags in Fig 1 are the corresponding Twitter accounts (e.g., https://twitter.com/mercuriovalpo)) that represents *El Mercurio de Valparaíso*, one of the oldest newspapers in Chile currently in circulation. This newspaper is part of a big conglomerate (*El Mercurio S.A.P*) that owns more than 20 news papers and several radio stations, among other broadcast media (such as magazines, TV cable, etc.). The regional newspaper *Soy Coronel* (soycoronel), on the bottom, is also part of this group. In fact, 11 regional newspapers of *El Mercurio S.A.P* are within these 26 and are all clustered bottom-right. As we mentioned earlier, the *El Mercurio*’s newspapers are popularly perceived as right-wing conservative.

*La Tercera* (latercera), is owned by *Copesa S.A*., which is *El Mercurio*’s closest competitor. These two companies have a so-called news media duopoly. *La Tercera*, also in the lower-right, but closer to the center of the group, is thought to be moderate-conservative [45]. *El Mostrador* (elmostrador) is an on-line newspaper with a perceived orientation to progressivism [46].

Finally, we want to mention *La Nación* (nacioncl) since it is a newspaper that currently only publishes its online edition and is partially controlled by the government. This newspaper appears in the top region of the *personal* dimension. Compared to the other 25 news outlets, this one appears as the most progressive on personal issues. This score is due to a series of populist reforms promoted by the government during the observed period (i.e. therapeutic marihuana legalization, decriminalization of abortion, anti-xenophobic campaigns, promote voluntary enlistment of women to the military service, etc.).

The perceived bias assigned to the rest of the ***26ers*** is shown in Table 4. Notice that many outlets in that list are perceived as *Right-wing, conservative*. There is also a group labeled as *Libertarian*. None of those outlets’ *PolQuiz* automatic classification correspond with their popular recognized leaning. Our hypothesis is that this mismatch is produced by a lack of contextualization: if we look at the range of scores obtained by the automatic method, we notice that they are confined to a fraction of the entire space. In order to investigate our proposition, we normalized the original scores by making the range of observed values our entire universe. We discuss these results in the next section.

**Table 4:** Perceived bias of the *26ers* extracted from Wikipedia.

Id	Name	Owner	Political alignment
1	adnradiochile	Grupo Prisa	International
2	biobio	Bío-Bío Comunicaciones	Independent
3	cooperativa	Co. Chilena de Comunicaciones	Christian democracy
4	latercera	Copesa	Classical liberalism
5	mercuriovalpo	El Mercurio	Right-wing, conservative
6	publimetrochile	Grupo metro	International
7	emol	El Mercurio	Right-wing, conservative
8	soyarauco	El Mercurio	Right-wing, conservative
9	soyconcepcion	El Mercurio	Right-wing, conservative
10	soycoronel	El Mercurio	Right-wing, conservative
11	soyquillota	El Mercurio	Right-wing, conservative
12	soysanantonio	El Mercurio	Right-wing, conservative
13	soytalcahuano	El Mercurio	Right-wing, conservative
14	soytome	El Mercurio	Right-wing, conservative
15	dfinanciero	Grupo Claro	Right-wing, conservative
16	el_ciudadano	Red de medios de los pueblos	Libertarian
17	elmostrador	La Plaza	Libertarian
18	tele13_radio	Grupo Luksic & PUC	—
19	el_dinamo	Ediciones Giro Pais	Christian democracy
20	nacioncl	Estado de Chile	Left, Liberal
21	pinguinodiario	Patagónica Publicaciones	—
22	soychillan	El Mercurio	Right-wing, conservative
23	soycopiapo	El Mercurio	Right-wing, conservative
24	soyvaldiviacl	El Mercurio	Right-wing, conservative
25	soyvalparaiso	El Mercurio	Right-wing, conservative
26	t13	Grupo Luksic & PUC	—

The list is sorted by the perceived bias. Outlets with an unclear Political Alignment (shadowed rows in the table) were left out of the analysis.

### Relative positioning

**Fig 2:**
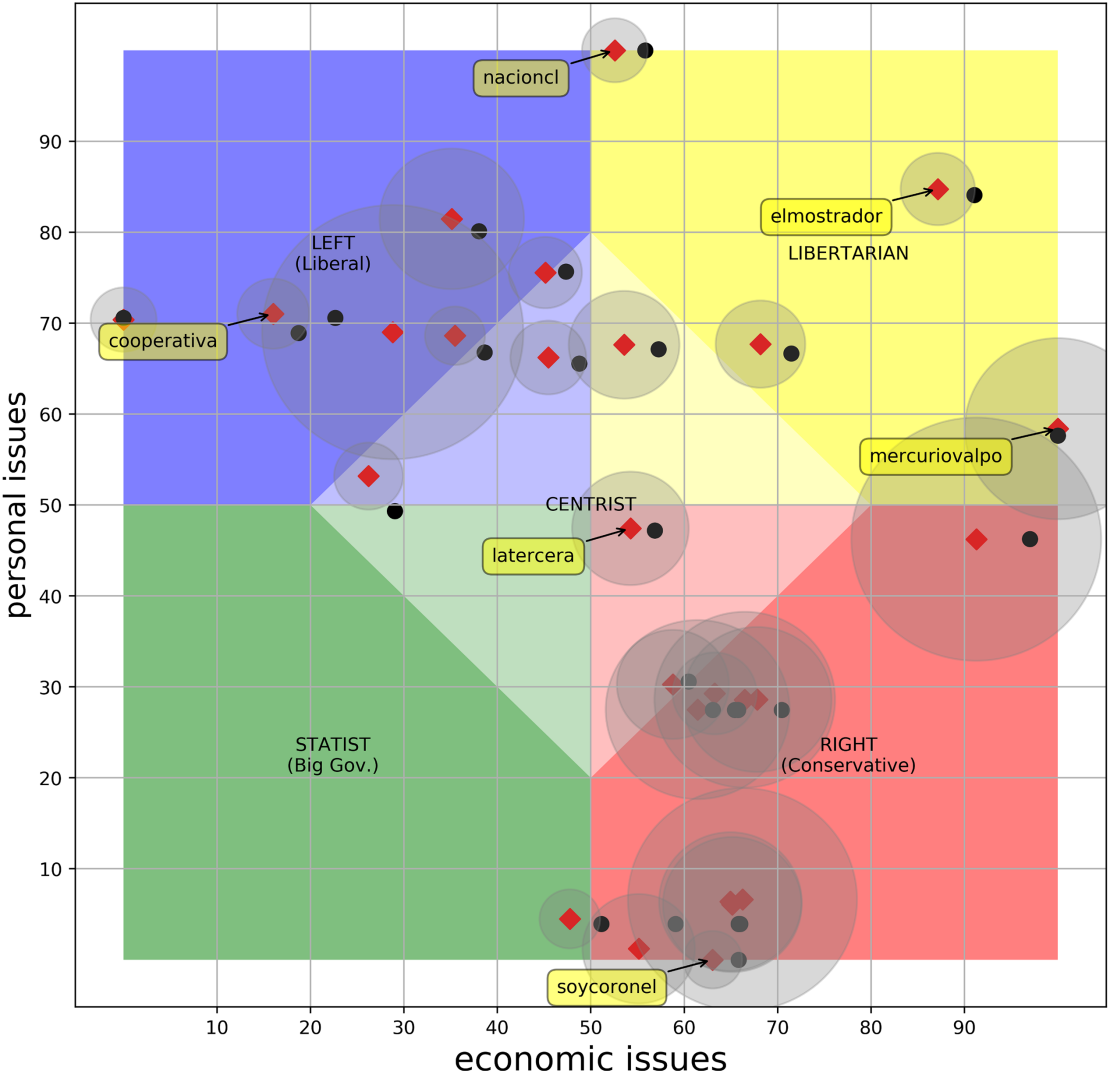
Relative position of the *26ers*. The dots represent the *relative* positions obtained by normalizing the PolQuiz scores. The blue dot shows the average for relative positions. Diamonds represent the score on each dimension as the average over 20 repetitions, leaving out each time a random 5% of the documents. The gray circles around diamonds are the 95% confidence interval.

We applied normalization to contextualize the political leaning of the outlets to the reality of Chile. We normalized the scores on each axis in the range [0, 100]. Now our entire positioning universe is determined by the scope defined by the Chilean media. Fig 2 shows the relative position of the *26ers* as black dots. Now each outlet is positioned *relative to the others*. These new position are much more inline with how people think of the leaning of these outlets.

This result shows that the “bias” should not be a categorical measure. Media bias comes embedded in a geopolitical news context determined by other outlets in the region. In other words, some bias is inherent to the media, but how biased they are (and on what direction they lean) will depend upon a comparison to other media in the same context. But, more importantly, *our own perception of the bias seem to be adjusted and limited by the political space defined by the news that we receive as a population*. This phenomenon has important socio-politic implications (such as the possibility of artificial displacement of the center for political purposes), but we leave to social scientist further considerations on these matters.

We noticed that even with the normalized scores, the Chilean media is not balanced. For our statistical analysis we treated each axis independently, so we could work with values in only one dimension. We conducted a one-sample Student t-test (the QQplot and the histogram suggested normality was a reasonable assumption) for each dimension (economic and personal) to test if the mean score was significantly different from 50 (the assumed unbiased score). We used, for each dimension, the scores of those news outlets for which we were able to answer at least one question on that dimension. For the economic dimension, there is a significant bias, *t*(254) = −10.93, *p* < .001, with a leaning to the left-wing (*M* = 40.28, *SD* = 14.21). In the personal issues the bias is lower, but still is statistically significant, *t*(190) = −2.10, *p* < .05, with a leaning to the conservative side (*M* = 47.42, *SD* = 16.98).

Once again, we think the slight left-wing bias in the economic issues might be explained, at least in part, by the political context of Chile during the observed period (see Section *The influence of government orientation on the media landscape*). On the personal issues dimension, we can also see some bias, although less prominent, tending to the conservative end of the spectrum. Other possible factors that might contribute to the observed tendency are the unavoidable bias introduced by the quiz itself and the presented methodology. The *PolQuiz* has been criticized as being biased by using leading questions, favoring libertarian results and imposing the libertarian definition of freedom [47]. For our part, we tried to minimize the bias in the methodology by making a conscious collective selection of the initial keywords for each query, but there is always room for interpretation of the questions. Despite the alleged bias, we have shown that the quiz can differentiate outlets with opposite points of view in both dimensions and also that the automatic classification is in accordance with the widespread perception of the tendency displayed by the main outlets. This means that either the bias introduced by the appliances in the methodology is not significant or it is representative of the predisposition showed by the population that we are considering in our study.

#### Stability of the results

In order to find out the stability of the observed bias with respect to changes in the obtained evidence (i.e. the collected tweets), we repeated the scoring steps 20 times. Each time we leave out 5% of the tweets selected at random, while maintaining the original distribution of documents per question. Each time, we measure the average score of the news outlets for which we were able to answer at least one question in the corresponding dimension. In the *economic* issues, we could observe a consistent bias to the left (*M* = 40.45, 95% *CI* [36.91, 43.99]). On the other hand, the *personal* dimension, although it is also leaning to one side, is much closer to the center of the spectrum (*M* = 46.89, 95% *CI* [43.99, 49.79]). Fig 2 shows a similar analysis, but at an individual level in the ***26ers***. The mean for each individual outlet (diamonds in Fig 2) stays close to its original position, and each newspaper can be located in a relatively small neighborhood with high confidence, meaning that there are no drastic changes compared to the previous classification.

The relatively low impact of leaving out data in the positioning process indicates that the results are not very sensitive to change and not influenced by only a small number of tweets.

#### Contextualizing the PolQuiz

**Fig 3:**
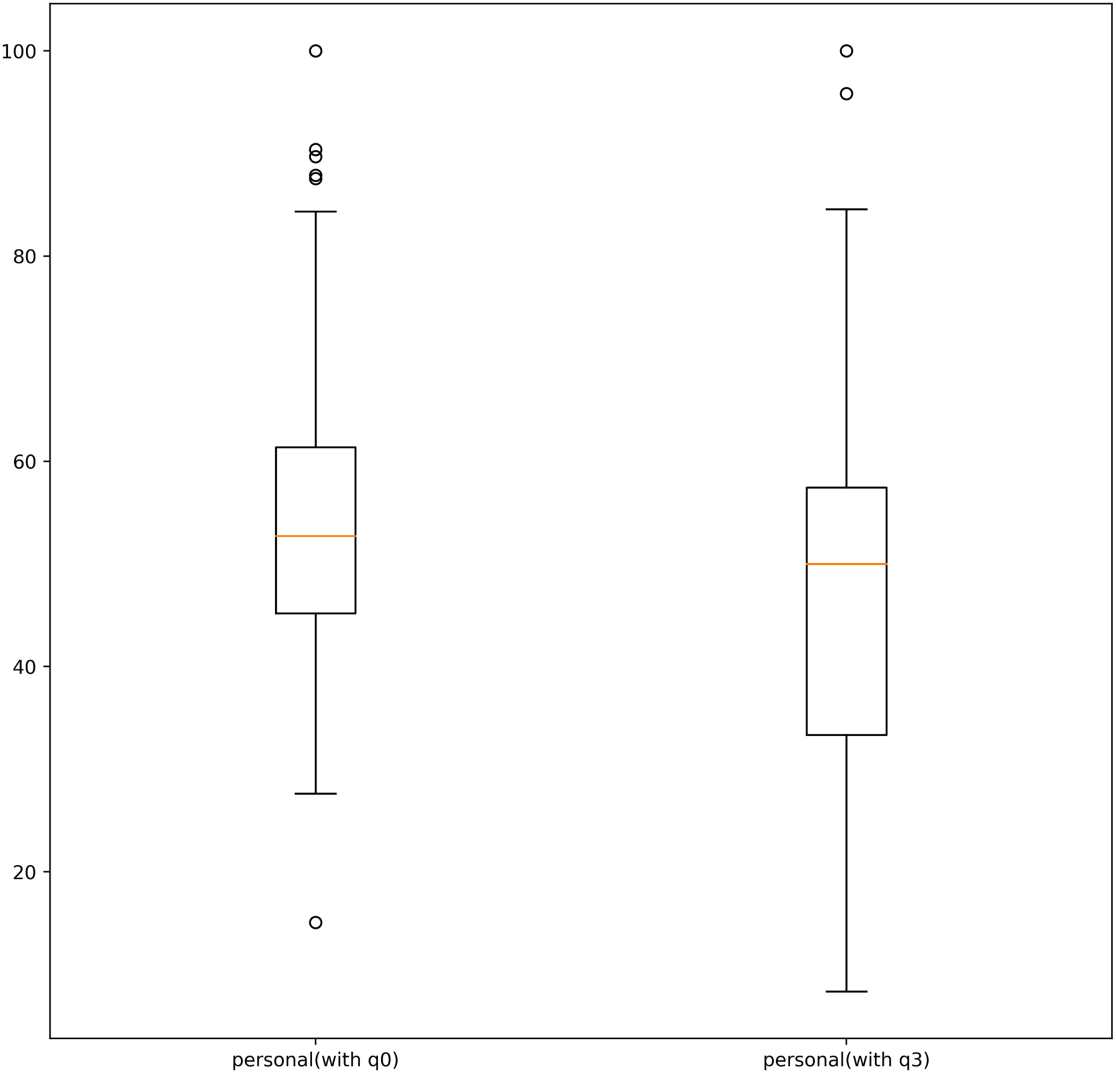
Scores before and after replacing q3 by q0. These are the scores of news outlets for which we were able to answer at least one question in the corresponding dimension.

We noticed that some of our queries, particularly in the personal issues dimension, returned only a small number of documents (e.i **q2** and **q3**). This is because of lack of interest or too few relevant events related to the corresponding topics during the observed period. We think that a way to counteract this environmental/circumstantial effect is to substitute the respective questions or to increase the number of questions. As a proof of concept, we repeated our analysis using **q0** as a replacement for question **q3** (related to laws concerning sex between consenting adult, see Section PolQuiz). We replaced **q3**, because it was the one with the lowest number of retrieved documents. This substitution increased the number of news outlets with at least one answer. There is now a stronger statistical effect for the personal issues dimension, *t*(239) = 3.54, *p* < .001. Interestingly, this dimension now leans to the more liberal end of the spectrum (*M* = 53.57, *SD* = 15.63) (see Fig 3).

**Fig 4:**
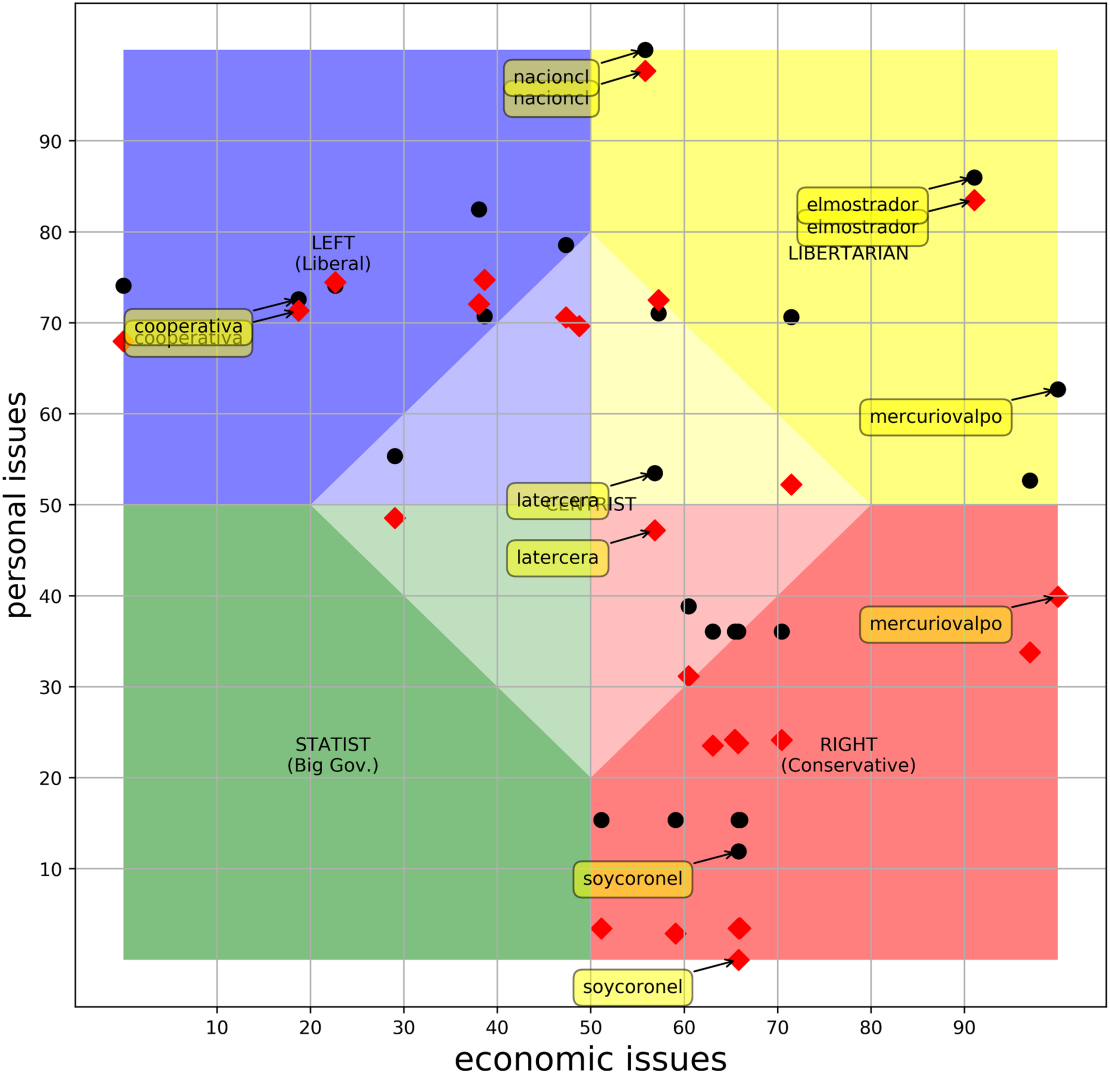
Relative position of the *26ers*. Dots represent the scores with q3. Diamonds represent the scores with q0.

In Fig 4 we plot the scores of the ***26ers*** in the original quiz (dots) and the adapted quiz (diamonds). Note that the difference in scores between the quiz with **q3** and the quiz with **q0** is considerably larger (with a negative difference) for outlets in the right/conservative quadrant. This is expected and validates the model.

### The influence of government orientation on the media landscape

As we mentioned earlier, we think that the overall behavior of the media, both in terms of their original/absolute position and the relative balance, is determined, at least in part, by the current government political alignment. This assumption is suggested by the absolute positions as shown in Fig 1 and supported by some models concerned with the political-economy of the mass media. The *Propaganda Model* [2] describes the political elite, and the government in particular, as a very influential actor. According to the model, either by millionaire advertising contracts, controlling the sources or generating flack against opposed views, the government always tries to control the discourse. The *Media Capture* model [48] also presents the government as an important factor on the news selection and publishing process. For an in-depth survey on the political economy of the mass media see also [49].

In this section we investigate if our *PolQuiz* methodology is able to give evidence on the influence of the political ruling class over the mass media behavior. For our analysis we apply the political quiz to the same set of news outlets, but using a different time frame. We collected the tweets in the analogous period of the previous administration. Since the previous government (lead by Sebastian Piñera) had a different political alignment (right-wing conservative), we should be able to see a shifting in the position of the outlets in between the two governments.

Using the Advanced Search from Twitter we collected 832,223 tweets, from 283 news outlets, published in the period from Oct 1st, 2010 to May 31st, 2011. This data set contains only tweet published by these outlets (no retweets are included). After applying the queries, our final dataset contains 16,176 tweets related to the issues addressed in the ***PolQuiz***.

**Fig 5:**
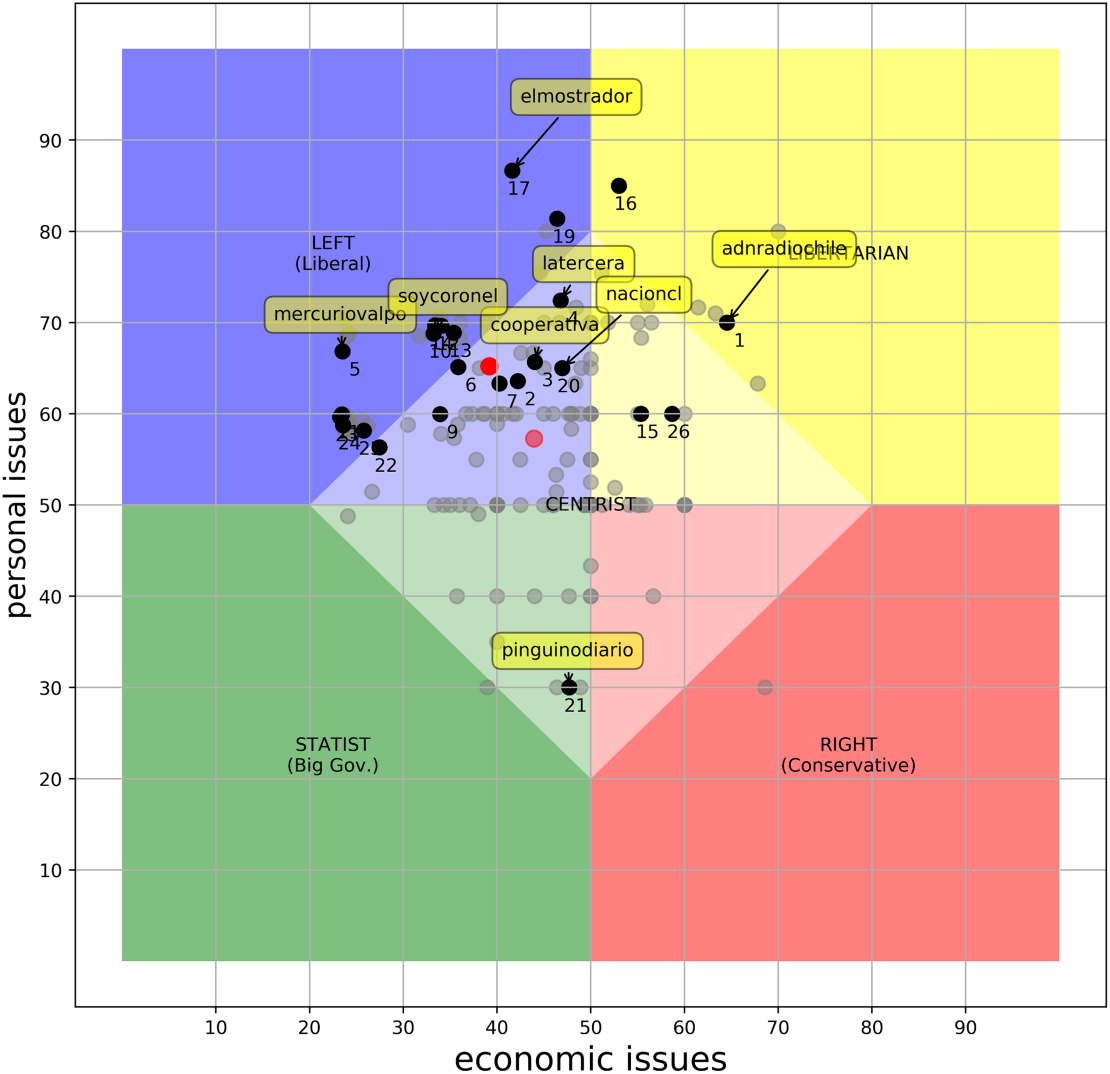
Absolute positions of the Chilean media during the right-wing/conservative government. The chart shows the position of the outlets for which we were able to answer at least one question. Dark dots represent the *26ers* subset. The solid red dot shows the average position for the *26ers*.

In Fig 5 we can see the absolute position of the 186 outlets for which we were able to answer at least one question of the *PolQuiz* using the tweets published during the previous government. In comparison with Fig 1 it is easy to notice that the entire context of the media as a whole is more to the center of the chart, or seem from the point of view of the current state of the media, it is more to the right and more conservative. This behavior might be due to the main topics being discussed (e.g., tax reforms vs. free high education), but ultimately this indicates that in a way or another the government is playing its role and it is dominating the discourse that prevails in the media.

We also compared the relative positions of the ***26ers*** in both governments (there are two of them that were not yet created in the first time slot). The Kendall’s Tau-b correlation for the economic dimension between the two periods is *τ*_*b*_(23) = −0.3461 (*z* = −2.37, *p* = .0178). This shows that there is association between the two time periods (i.e. we can reject the null hypothesis of independence), but there are quite a few inversions in the relative order. This is because the newspapers owned by El Mercurio stayed more or less in the same position while most of the others move from being right to El Mercurio’s to being left of their position (notice in Fig 5 that *mercuriovalpo* is at the left of the group). We mentioned before that *El Mercurio S.A.P* is the biggest media group in Chile, but now we can say that it is also the most stable in their editorial policy behaviour. This makes intuitively sense: their consolidated control of the market gives them more independence, and it makes them less susceptible to the government influence.

**Fig 6:**
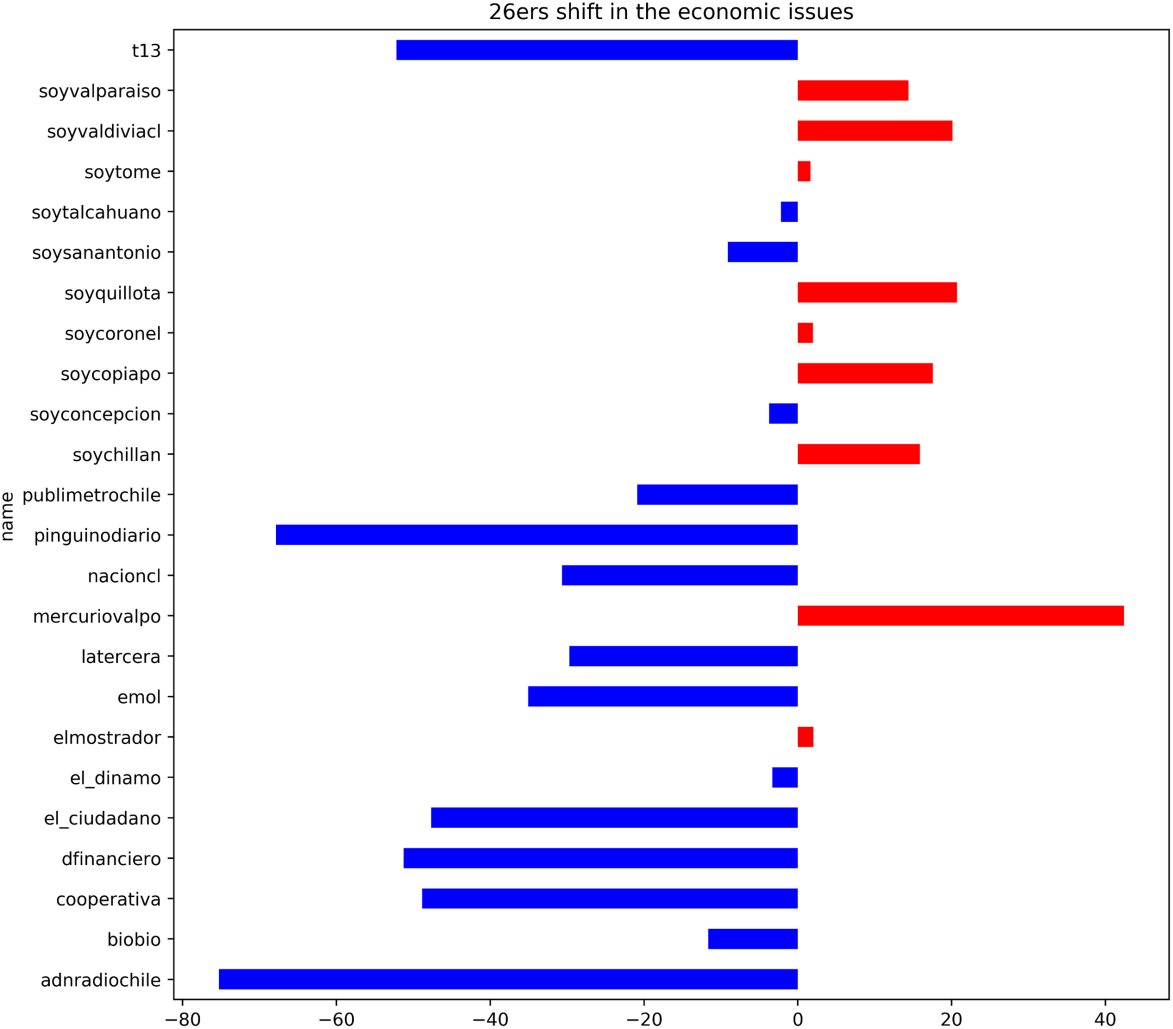
Shift in the position of the *26ers* for the economic dimension. The chart shows the relative shift in points from the conservative to the liberal government. A shift > 0 (red bars) means more to the right. A shift < 0 (blue bars) means more to the left left.

To study the individual behavior of the outlets, we also calculated the relative position of the outlets in one period with respect to the other period. For this we normalized the scores using the results from both periods combined. This allows us to see, in the overall context, the outlets transition over politically opposite regimes. Fig 6 shows how many points each of the ***26ers*** move within this context in the economic issues dimension. Notice that there is a tendency towards the left with the arrival of the left-wing government. Only some of the outlets owned by *El Mercurio S.A.P.* stayed in an approximately similar position or shifted to the right. *El Mercurio de Valparaiso* shows again to be one of the most important representatives for the right. Interestingly, the biggest movements to the left (in the same direction of the new government), come from outlets from which we were not able to find a clear popular perception or declare political leaning. Maybe is this flip-flopping what makes it so difficult for the public.

**Fig 7:**
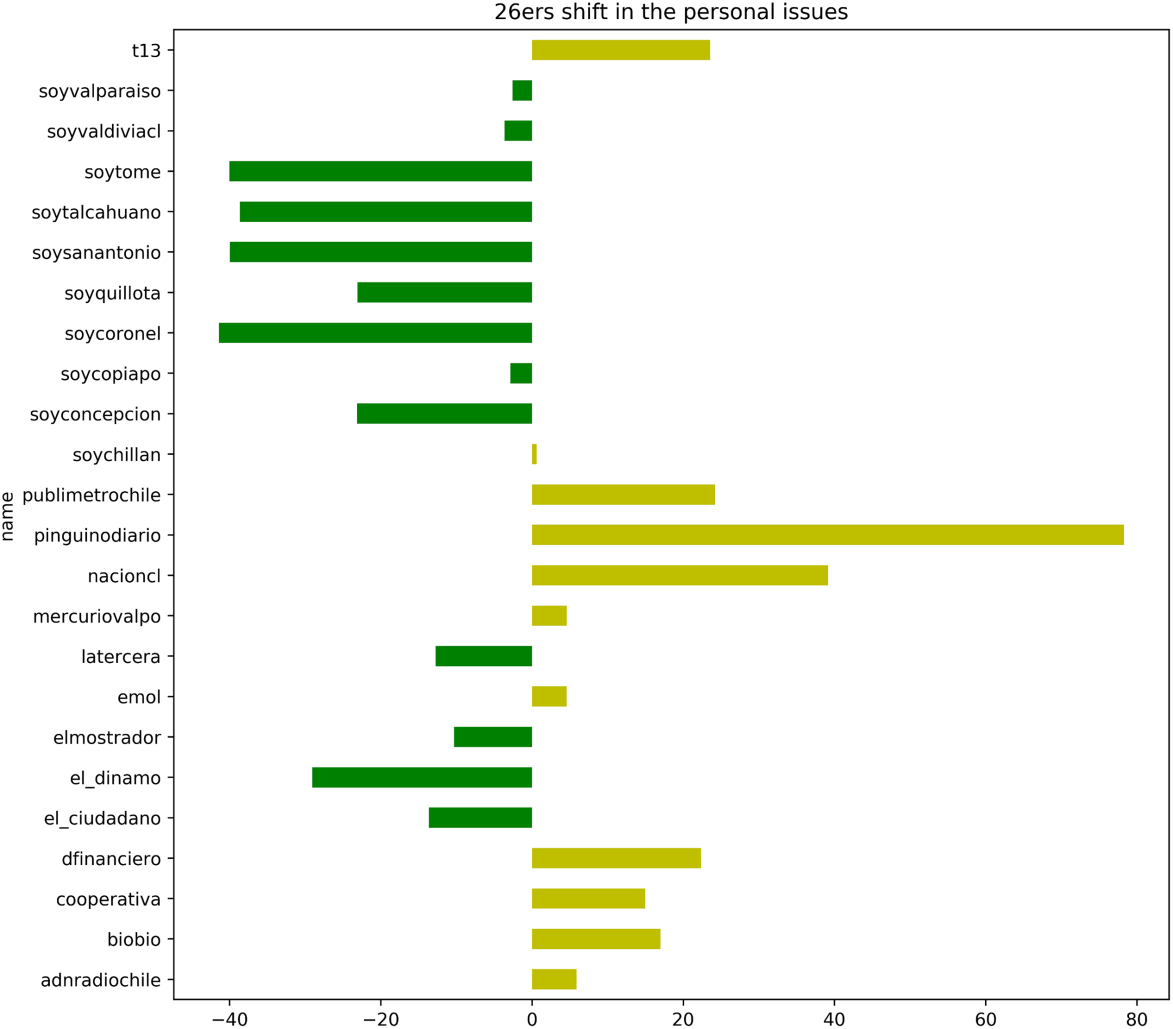
Shift in the position of the *26ers* for the personal dimension. The chart shows the relative shift in points from the conservative to the liberal government. A shift > 0 (yellow bars) means more liberal. A shift < 0 (green bars) means more conservative.

Fig 7 represents a similar individual analysis for the personal issues dimension. As in the results presented earlier, this dimension offers less information. The direction of the movements is more divided and seems to emphasize the known position of the outlets. Of particular interest is the outlet controlled by the government (*nacioncl*). This newspaper’s editorial policy seems to move (in both dimensions) to accommodate the current government. This behavior makes it a biased source of information, but a good point of reference to validate our model. Another point to notice is that, once again, the outlets without a clear perceived bias, consistently show the most significant shift in favor of the ruling side.

This empirical analysis of the behavior of the media over two politically different regimes show that, directly or indirectly, the government does successfully interfere in the news process. The case of Chile makes a good example because being only four years apart makes it harder to attribute these noticeable differences to other factors (such as a very different staff, ownership or editorial policy).

### Investigating the nature of bias using rank difference

The *PolQuiz* showed the existence of bias in Chilean media. In this section, we investigate the nature of this bias in terms of vocabulary used and entities mentioned in the different newspapers’ tweets (see Section Rank difference). We focused on the ***26ers*** and the topic of abortion. We selected the topic of abortion, as it is one of the most polarizing issues in our dataset. Nevertheless, this is used only as an illustration: to fully understand the nature of the bias and the media landscape, the decision makers or interested parties should conduct a similar analysis on each of the questions.

#### Topic bias based on named entities

We used the Stanford’s NE recognizer system [50] to extract the entities mentioned in the tweets related to the abortion issue. We compare the extracted entities against a list of politicians, public personalities and activist groups. For the list of politicians and their position in the abortion issue, we use the vote sessions in the house of representatives [51] and in the senate [52]. We manually labeled another 53 personalities and groups according to comments and events reported in the local news. The complete list *L*_*E*_ has 199 labeled entities. We labeled with −1 the politicians who voted against the abortion bill, and the public figures that were openly against the issue. Equivalently, we use + 1 for politicians and personalities in favor of the subject. We assign a 0 to the entities not included in our list. We will refer to these labels as the leaning of the entities (e.g. *leaning*(*entity*)).

After applying the rank difference method to the NE mention counts, we calculated a score for each outlet in function of the *τ*(*entity*) and the leaning of *entity* in the issue (for every *entity* mentioned more than once in the news). This final score of the outlet *o*_*i*_ is found using the Eq 2.

$$score\left(o_{i}\right)=\sum_{e\in L_{E}}(\tau (e)*leaning(e))/size\_of\left(L_{E}\right))$$ (2)

A low value in this score indicates that this outlet tends to mention with relatively high frequency entities with a conservative leaning and/or it tends to ignore those with a more liberal view.

As expected, outlets tagged as independent, libertarian and classical-liberal have higher scores (top 10 in the ***26ers***). Interestingly, within the top 10 we also find the outlets tagged as *International*, publimetrochile and adnradiochile, which means that they behave similarly to liberal outlets under the left-liberal government in office in 2016. According to our scores, all these top-scored outlets have comparably more mentions of entities with a liberal leaning than the rest of the outlets. To our surprise, the lower values (bottom 5 in the ***26ers***) are occupied by the outlets linked to parties in the ruling coalition (Christian democracy and Left-Liberal(nacioncl)). Apparently these outlets focus their tweets in negative reports of the opposition. For example, when we look at the rank-difference results for nacioncl, within the top-20 entities, only two refer to entities with a liberal leaning (‘President Michelle Bachelet’ and ‘Government’). To investigate more on this, we run a sentiment analysis on the most used bigrams. The results are presented in the next section.

#### Topic bias based on bi-grams

We again apply the rank difference method, this time using the bi-gram counts in the tweets relevant to the subject of abortion. Following the same strategy as before, we calculated a score for each outlet in function of the *τ*(*bigram*) and the sentiment calculated for *bigram* (for every *bigram* mentioned more than once in the news). For determining the sentiment of words and bi-grams we use the Spanish lexicon from [53]. This lexicon consists of a set of norms for valence and arousal for an extensive set of Spanish words. We found this to be one of the largest dictionaries in this language, and it includes items from a variety of frequencies, semantic categories, and parts of speech, including conjugated verbs. We weighted each word with its mean valence (we assigned the neutral value 5 for words not present in the dictionary). The weight of the bi-grams is the average of the weight of their composing words. To calculate *τ*(*bigram*) we use a formula equivalent to that shown in Eq 2. Accordingly, we give a similar interpretation to these scores. That is, a high value indicates that this outlet tends to convey mostly positive sentiments with the bi-grams used with relatively high frequency and/or avoid using negative sentiments when referring to the issue of abortion. For example, *elmostrador*, with the highest score, has as a frequently use bi-gram “proyecto aprobado” (tr. “project approved”—referring to the bill). This bi-gram is classified as positive by the sentiment analyzer, so it will add to the score. On the other hand, this same outlet has “injusticia gobierno” (tr. “government injustice”) as a totally ignored bi-gram. Since the bi-gram is assigned a negative sentiment and the rank-difference is also negative, the bi-gram will also add to the score of the outlet, pushing it to the liberal side. Following the same reasoning, an outlet with a very low score can be understood as an outlet that uses predominantly negative words with relatively high frequency.

When we analyze the scores of the ***26ers***, we notice that *nacioncl* (controlled by the government) has the lowest score. This, together with the previous NE analysis, confirms the theory that this outlet focuses in tweeting negative reports of the opposition, at least for the abortion issue. Most of the others outlets show the expected behavior, with conservative in the lower half of the ranking (i.e. lower scores) and liberals in the higher positions.

The question that follows is if the bias that we are seeing with the *PolQuiz* and describing with the Rank Difference is perceived in the same way through the popular wisdom. We help answer this question in the next section.

### Survey results

For the survey described in Section Survey, we collected 372 answers from 54 unique Chilean users on how they perceive the bias on the topic of abortion in the different Chilean newspapers. Since this was an open and anonymous online survey, we do not have any demographic data on the users, but the IP addresses indicate we have a good representation of different regions of the country. We received between 11 and 19 answers for each of the ***26ers*** (M: 14.31, SD: 2.07). We carried out 10 Fleiss’ kappa measurements; each time we selected 10 ratings at random per outlet (subject). This shows a fair agreement in the answers (M: 0.2253, SD: 0.0167). In Table 5 we show the ***26ers*** and their corresponding “Perceived bias” (see Section Survey). The political alignment information shown in the table is again our ground-truth.

**Table 5:** Results from popular survey for the *26ers*.

Id	Name	Owner	Political alignment	Perceived bias	Personal issues
21	pinguinodiario	Patagónica Publicaciones	—	-66.67	39.18
24	soyvaldiviacl	El Mercurio	Right, conservative	-66.67	-50.49
22	soychillan	El Mercurio	Right, conservative	-57.14	-50.55
25	soyvalparaiso	El Mercurio	Right, conservative	-43.75	-51.81
8	soyarauco	El Mercurio	Right, conservative	-42.86	-51.27
12	soysanantonio	El Mercurio	Right, conservative	-30.77	-92.98
13	soytalcahuano	El Mercurio	Right, conservative	-30.77	-92.98
18	tele13_radio	Grupo Luksic & PUC	—	-28.57	52.42
9	soyconcepcion	El Mercurio	Right, conservative	-25.00	-94.09
14	soytome	El Mercurio	Right, conservative	-25.00	-92.92
7	emol	El Mercurio	Right, conservative	-25.00	-0.59
10	soycoronel	El Mercurio	Right, conservative	-23.53	-100
11	soyquillota	El Mercurio	Right, conservative	-18.18	-92.92
15	dfinanciero	Grupo Claro	Right, conservative	0.00	42.57
5	mercuriovalpo	El Mercurio	Right, conservative	21.43	-51.81
2	biobio	Bío-Bío Comunicaciones	Independent	23.53	6.91
6	publimetrochile	Grupo metro	International	25.00	47.50
17	elmostrador	La Plaza	Libertarian	26.32	70.95
19	el_dinamo	Ediciones Giro Pais	Christian democracy	29.41	-30.79
4	latercera	Copesa	Classical liberalism	33.33	-3.38
1	adnradiochile	Grupo Prisa	International	37.50	52.98
16	el_ciudadano	Red de medios de los pueblos	Libertarian	37.50	44.54
23	soycopiapo	El Mercurio	Right, conservative	38.46	-36.21
3	cooperativa	Co. Chilena de Comunicaciones	Christian democracy	57.14	46.04
26	t13	Grupo Luksic & PUC	—	57.14	48.45
20	nacioncl	Estado de Chile	Left, Liberal	63.64	100

The list is sorted by the perceived bias. Outlets with an unclear Political Alignment (shadowed rows in the table) were left out of the analysis.

Results show that there is a perceivable difference in the language used by the outlets in both sides of the spectrum. Note that, based on the rank difference of bi-grams, the users were able to collectively classify the outlets with over 90% precision (We are not taking into account those for which we could not find a political alignment or those that belong to international groups). Our positioning of these outlets in the adapted *PolQuiz* has also a good agreement with the direction of the Perceived bias (80%).

To evaluate the relative positions of the outlets in our *PolQuiz*, we calculated the number of inversions with respect to the ranking of the outlets in the perceived bias. The Kendall’s Tau-b coefficient between the two rankings is *τ*_*b*_(21) = 0.4203 (*z* = 2.66, *p* < .01). Even though the popular perception resulting from the survey can not be seen as ground-truth for the relative positioning of the outlets, it is important to notice that our results show a good correlation with the intuition of the public. As a future work, we aim to add some other content features (e.g., leaning of the named entities) to the polarity classification of the tweets as these may help to refine the relative positioning found by our model.

To summarize, we have shown that reported political alignment is highly correlated with the *PolQuiz* results as well as with the bias, as perceived by the general audience. This implies that existing bias has a noticeable influence on how controversial issues such as abortion are reported in the media.

## Conclusions

In this paper, we presented an automatic approach for estimating the political bias of news outlets in Chile, exploiting the well-known and widely used “The World’s Smallest Political Quiz”. We empirically confirmed the estimation results and showed that they are stable with respect to evolving data. We have demonstrated the benefits of adapting questions to the local context. Furthermore, we showed that our model is able to discover the relative political context that regulates the perceived bias of the media. Building upon the *PolQuiz* results, we investigated the nature of this political bias and found this to exist in the chosen vocabulary and the entities covered by the newspaper. We also conducted a survey, of which the results confirm that political bias in newspapers has an impact on how controversial topics are covered and that the general audience does notice this bias. Our methodology does not make too many assumptions about the underlying system. The way it is designed could be applied to any Western culture. Our system can deal with any number of outlets, can compare relative quantitative positions, can show empirical evidence of consistent bias, and can partially explain the source of these tendencies.

Finally, our methodology contributes as empirical evidence of the media capture in modern “democratic” societies. Since most people expect the media to report a fair an unbiased account of the events, we think the outlets behavior should be analyzed and take into consideration in, for example, recommending systems for a real diversity of information.

In summary, the results indicate that the political orientation of the media in Chile is in line with and follows the political orientation of the government. Even though relative differences in bias or orientation between individual news outlets can be observed, public awareness of the bias of the media landscape as a whole appears to be limited: our own perception of the bias seem to be adjusted and limited by the political space defined by the news that we receive, which on its turn is largely defined by governmental politics.

We believe it is important to be aware of shifts, alignments and discrepancies in bias and political orientation within the government, the population and the media, as misconceptions regarding real or perceived bias may have unexpected or negative effects.

As a future work we are interested to see what is the most accurate way to score the missing answers. Since “coverage” is a form of bias [4], perhaps the outlet is not being *neutral* by not mentioning a specific subject. Even when the decision of which stories/events are newsworthy is subjective and depends on the editorial strategy [6], there are some events that are very relevant in the national context and are covered for the majority of the media. So, a complete silence of a news outlet on such an event may be interpreted as something other than neutrality.

**Fig 8:**
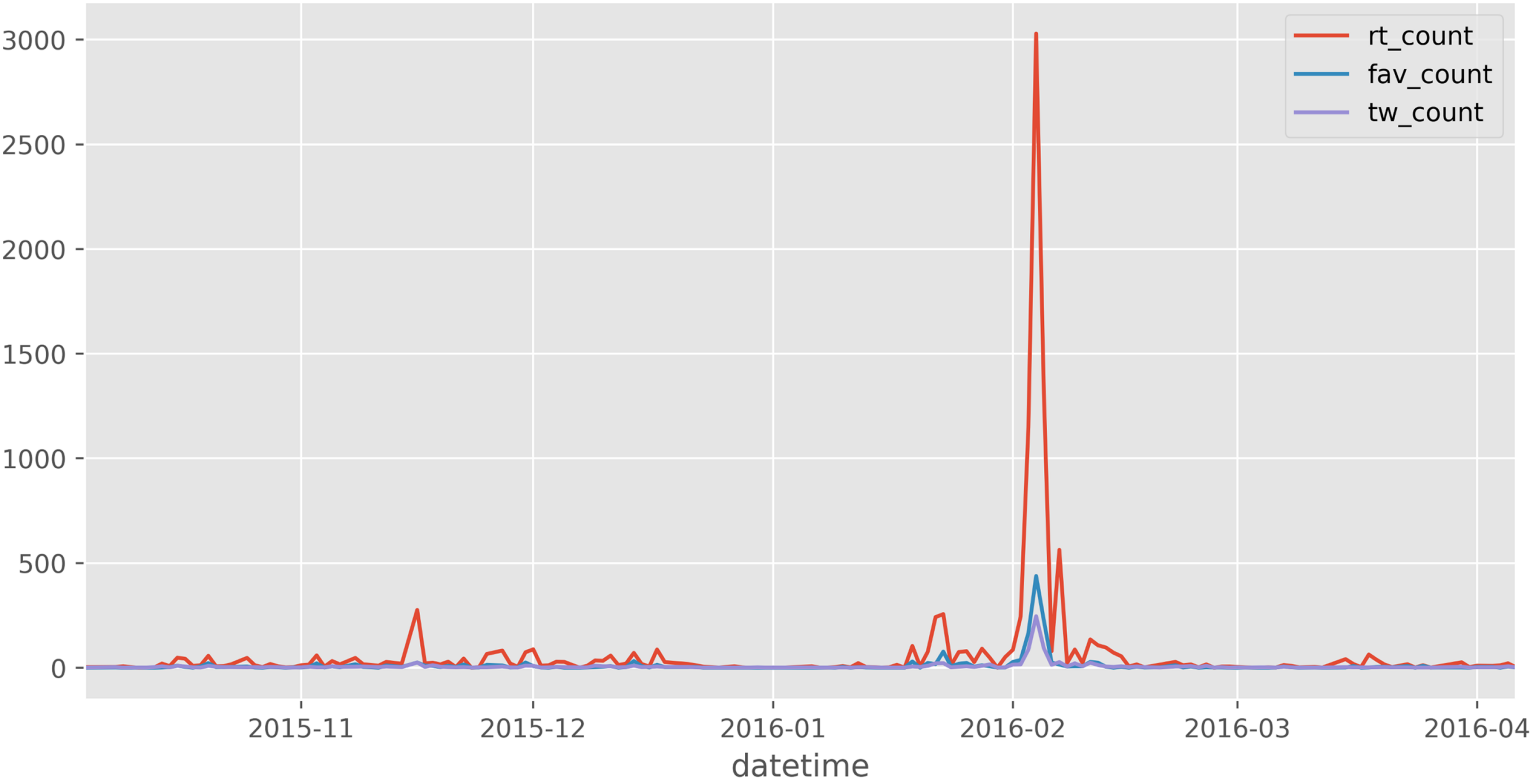
Counting tweets and re-tweets for q7 under president Bachelet.

For example, question **q7** is about international free trade. Taking the number of tweets and re-tweet as an indicator of important events [3], we can see in Fig 8 that this topic has had at least one major event during this period. This event was the ascription of Chile to the Trans-Pacific Partnership (TPP) signed by the country on Feb 3th, 2016. Despite the magnitude of the event, only 135 out of 198 newspapers with a section on politics mentioned it. A plausible cause is that the other news outlets decided not to report about this event, in other words ‘bias by omission’.

We show that a careful selection or update of the questions may lead to a significant improvement in the results. If we have an inside understanding of the socio-economic environment from where the news are being collected, then we could replace the questions to capture more relevant topics. In this sense, we could benefit from advances in systems that focus on identifying controversial topics in social media [54]. On the other hand, if we do not have any intuition on the news collected, then we can accumulate the new questions so we can widen the spectrum of topics and have a better chance of capturing relevant events/discussions with our queries.

In the future, it would be interesting to compare the results of this paper to a similar analysis conducted over full-text articles published by the same news outlets. As discussed, this will require more sophisticated NLP tools and more human supervision, but it could shed some light on the similarities and differences between traditional media and social media.

For individuals as well as for society as a whole it is important to recognize and understand media bias that are shaped through underlying general political or socio-economic orientations. As we have shown in this paper, these general tendencies have a clear and noticeable effect on the way concrete topics are covered and commented upon, and therefore should be investigated and published.
